# Mirikizumab is associated with improvements in bowel urgency and quality of life in moderately-to-severely active ulcerative colitis: 28-week phase 3b results from the LUCENT-URGE trial

**DOI:** 10.1093/crocol/otag107

**Published:** 2026-09-24

**Authors:** Millie Long, David Laharie, Gerhard Rogler, Seyedehsan Navabi, Aisha Vadhariya, William J Eastman, Tian Tian, Alison Potts Bleakman, Alessandro Armuzzi

**Affiliations:** Division of Gastroenterology and Hepatology, University of North Carolina, Chapel Hill, NC, United States; CHU de Bordeaux, Centre Medico-Chirurgical Magellan, Hospital Haut-Lévêque, Gastroenterology Department, Université de Bordeaux, INSERM CIC 1401, Bordeaux, France; Department of Gastroenterology and Hepatology, University Hospital Zurich, University of Zurich, Zurich, Switzerland; United Medical Doctors, Gastroenterology, Murrieta, CA, United States; Eli Lilly and Company, Indianapolis, IN, United States; Eli Lilly and Company, Indianapolis, IN, United States; Eli Lilly and Company, Indianapolis, IN, United States; Eli Lilly and Company, Indianapolis, IN, United States; IBD Center, IRCCS Humanitas Research Hospital, Rozzano, Milan, Italy; Department of Biomedical Sciences, Humanitas University, Pieve Emanuele, Milan, Italy

**Keywords:** bowel urgency, mirikizumab, quality of life, ulcerative colitis

## Abstract

**Background:**

Mirikizumab, an anti-interleukin-23p19 monoclonal antibody approved for ulcerative colitis (UC) and Crohn’s disease, was evaluated in LUCENT-URGE (NCT05767021), a phase 3b, open-label study assessing its effect on bowel urgency (BU) through week (W)28 in adults with moderately-to-severely active UC, using novel BU measures and validated quality of life (QoL) instruments.

**Methods:**

Patients with UC experiencing BU (baseline severity ≥3 on the urgency numeric rating scale [UNRS]) received 300 mg mirikizumab intravenously at W0, W4, and W8, then 200 mg subcutaneously at W12 and every 4 weeks up to W24. Improvements in QoL and patient-reported outcomes were assessed from baseline to W12 and W28.

**Results:**

Among 172 patients, 58.1% achieved clinically meaningful improvement (≥3-point reduction in UNRS), and 37.8% reached BU remission (rounded UNRS ≤ 1) by W28. Inflammatory Bowel Disease Questionnaire (IBDQ) scores improved by 56.4 points, with 52.3% of patients achieving IBDQ-remission (IBDQ ≥ 170) and 65.7% achieving IBDQ-response (≥16-point increase) at W28. Absorbent product use for BU dropped from 55.1% at baseline to 29.6% at W28. Patients reporting no sexual activity avoidance (SAA) due to UC symptoms rose from 21.1% to 51.4%. Activity impairment, Wexner Incontinence Score, and anxiety symptoms also showed improvements.

**Conclusion:**

The majority of mirikizumab-treated patients achieved clinically meaningful BU improvement over 28 weeks. In addition to QoL improvement, patients experienced reduced disruption of daily activities, with meaningful reductions in SAA and use of protective aids due to BU. Achieving BU remission or clinically meaningful improvement with mirikizumab was associated with improvements in patients’ relevant UC symptoms and overall QoL.

## Introduction

Ulcerative colitis (UC) is a chronic, immune-mediated inflammatory bowel disease (IBD) of multifactorial etiology, characterized by relapsing and remitting inflammation of the colonic and rectal mucosa, leading to ulceration and bleeding.^1^^,^^2^ Common symptoms of UC include diarrhea, rectal bleeding, abdominal pain, and bowel urgency (BU). These clinically important symptoms interfere with patients’ daily functioning, restrict social and professional engagement, and contribute to emotional distress and diminished quality of life (QoL).^2–5^

In recent years, UC management has evolved toward a more comprehensive paradigm that emphasizes not only clinical and endoscopic remission, but also improvements in patient-reported outcomes (PROs) and health-related QoL (HRQoL).^6^^,^^7^ This approach reflects broader understanding of the multifaceted burden of UC, including its psychosocial dimensions.^8^

Among the most distressing symptoms of UC is BU, the sudden and immediate need to have a bowel movement.^9–11^ Affecting over 80% of patients, with nearly half experiencing it daily,^12^ BU is associated with reduced deferral time (time the patient can defer defecation after the desire to evacuate), fear of fecal incontinence, social withdrawal, sexual activity avoidance (SAA), and reliance on protective aids (pads, diapers, etc.), all contributing to diminished QoL. Moreover, fear of BU may cause patients to avoid leaving the house even for grocery shopping,^9^ increasing packaged/processed food consumption and negatively affecting IBD management.^13^ However, despite its prevalence and impact, BU has historically been underrepresented as a clinical trial endpoint.^14^ Emerging evidence suggests that improvement in BU is correlated with better clinical outcomes and enhanced HRQoL.^15–18^

Mirikizumab, a selective monoclonal antibody targeting the p19 subunit of interleukin-23 (IL-23), has been approved for the treatment of both UC and Crohn’s disease in adults.^19^ The LUCENT clinical trial program (LUCENT-1 [NCT03518086], LUCENT-2 [NCT03524092], and LUCENT-3 [NCT03519945]) has previously demonstrated its sustained efficacy and safety in patients with moderately-to-severely active UC.^20^^,^^21^ Notably, in LUCENT-1 and LUCENT-2, mirikizumab demonstrated significant benefits in improving BU, assessed using the single-item, patient-reported Urgency Numeric Rating Scale (UNRS), with 65% of patients achieving clinically meaningful improvement (CMI, ie, ≥3-point reduction from baseline in the UNRS score) in BU and around 43% achieving BU remission (rounded UNRS ≤1) at week 52. ^22^ These improvements were accompanied by meaningful gain in QoL.^23^

Building on these findings, the LUCENT-URGE (NCT05767021) study was designed to further evaluate the impact of mirikizumab on BU using novel BU-specific measures, alongside validated instruments assessing HRQoL and work productivity in adults with moderately-to-severely active UC and current BU. Here, we report findings from this study, focusing on the effect of mirikizumab on QoL outcomes and the association between improvements in BU and QoL measures.

## Methods

### Study design and patients

LUCENT-URGE (NCT05767021) was a multicenter, phase 3b, open-label, single-arm clinical trial conducted over a 28-week period. The study design from LUCENT-URGE has been described previously.^15^ Briefly, the study included 4 sequential periods: a 28-day screening period, a 12-week intravenous (IV) treatment period, a 16-week subcutaneous (SC) treatment period, and a post-treatment follow-up period. Patients received 300 mg IV mirikizumab at weeks 0, 4, and 8, followed by 200 mg SC mirikizumab at weeks 12, 16, 20, and 24. Eligible patients were offered the option to enter a continued access period.

Eligible patients were adults with moderately-to-severely active UC who had experienced an inadequate response, loss of response, or intolerance to conventional therapies (corticosteroids or immunomodulators) or advanced therapies (biologics, Janus kinase [JAK] inhibitors, or sphingosine-1-phosphate [S1P] receptor modulators). Patients were required to have a confirmed diagnosis of UC for ≥3 months, a Modified Mayo Score of 4-9, an endoscopic subscore of ≥2, current BU (UNRS ≥ 3), and a surveillance colonoscopy negative for dysplasia or high-risk adenoma.

Key exclusion criteria included a diagnosis of Crohn’s disease, unclassified IBD, ulcerative proctitis only, past use of anti-IL-23p19 antibodies for any indication, or failure of >3 approved biologic/JAK/S1P therapies for UC. The detailed exclusion criteria have been described previously.

### Study outcomes and assessments

#### BU measures—patient-reported outcomes

BU measures described below were assessed using an electronic daily diary.

##### BU severity

BU severity was assessed using the UNRS, a validated, single-item, patient-reported instrument. Patients were asked to rate the severity of urgency to have a bowel movement in the past 24 hours using an 11-point NRS, ranging from 0 (no urgency) to 10 (worst possible urgency), in which higher scores indicate more severe BU. This PRO was previously validated in patients with UC.^12^ For analysis, the weekly average UNRS was calculated as the mean of daily values over a 7-day period. A ≥ 3-point reduction from baseline in the UNRS score was considered a CMI.^22^ UNRS remission is defined as rounded UNRS ≤1.^22^

##### BU frequency

BU frequency is a novel patient-reported, single-item measure of the number of urgent bowel movements reported in the past 24 hours. Responses were reported as integers representing the total number of urgency episodes. The weekly average BU frequency was calculated as the mean of daily values over a 7-day period.

##### Shortest weekly stool deferral time

Stool deferral time (SDT) is a novel patient-reported, single-item measure of the average duration a patient could delay defecation in past 24 hours. The shortest weekly SDT was defined as the minimum value reported during a 7-day period, with “no urgency” treated as the maximum delay. This approach was chosen to reflect the most limiting experience for patients and to provide robustness against outlier responses. Responses, reported in minutes or as “no urgency,” were categorized as follows: <1, 1–<2, 2–<5, 5–<15, ≥15 min or “no urgency.”^24^ The proportion of patients in each shortest SDT category results was reported.

#### Other PROs

Additional PRO measures were administered via an electronic tablet at baseline, week 12, and week 28 to assess key HRQoL domains from the patient’s perspective. QoL instruments included the Inflammatory Bowel Disease Questionnaire (IBDQ; total scores: 32–224),^25–27^ the Work Productivity and Activity Impairment: Ulcerative Colitis (WPAI: UC),^28^^,^^29^ the Wexner Incontinence Score (WIS),^30^ and the General Anxiety Disorder-7 (GAD-7).^31^ Detailed descriptions of these PRO measures are provided in Table S1. In addition, newly developed patient-reported QoL outcomes that are described below were assessed.

##### Absorbent product use for BU

The absorbent product use (APU) for BU is a single-item instrument assessing patients’ need to wear absorbent products (adult diapers, pads, or protection) due to BU over the past 2 months. Responses are rated on 5-point frequency scale: never (0), rarely (1; <1/month), sometimes (2; <1/week, ≥1/month), usually (3; <1/day, ≥1/week), and always (4; ≥1/day).

##### Sexual activity avoidance due to UC symptoms

SAA is a 2-question instrument that assessed patients’ avoidance of sexual activity, due to UC symptoms over the past month. Patients were asked to report the SAA frequency (range: 0–3) from “did not avoid” (0) to “always avoided” (3). If avoidance was reported, patients were asked to select reasons for avoidance from a list of 17 options (Table S2). Patients who selected “not applicable” at baseline for the SAA assessment were excluded from the analysis.

### Correlations between BU measures and PROs

Correlations between BU measures (BU severity, BU frequency, and SDT) and change in IBDQ, WPAI: UC, WIS, SAA due to UC symptoms, APU due to UC, and GAD-7 from baseline were analyzed at weeks 12 and 28.

Additionally, associations of “BU CMI” and “BU Remission” with changes in IBDQ and WPAI: UC and of IBDQ remission and response with changes in BU measures were evaluated.

### Statistical analyses

Descriptive statistics were used to summarize continuous variables, including mean with standard deviation (SD). Mean changes from baseline at weeks 12 and 28 were reported with corresponding 95% confidence intervals (CIs). Categorical outcomes were summarized using frequencies and proportions. Missing data and treatment discontinuations were addressed using non-responder imputation (NRI) for binary data and baseline observation carried forward (BOCF) for continuous data.

Correlation analysis was performed using Spearman’s rank correlation coefficient, selected for its robustness to non-normally distributed data, based on observed data excluding missing values at specific time points. Correlation strength was categorized as follows: 0.00–0.09 (negligible), 0.10–0.29 (very weak), 0.30–0.49 (weak), 0.50–0.69 (moderate), 0.70–0.89 (strong), and ≥0.90 (very strong).^32^ Associations between BU measures and PROs, as well as between IBDQ endpoints and BU measures, were analyzed using analysis of covariance (ANCOVA), with results expressed as standardized mean differences (SMDs). The level of association was categorized as follows: 0.20 to <0.50 (small), 0.50 to <0.80 (medium), and ≥0.80 (large). For binary endpoints, 95% CIs were calculated using the asymptotic method without continuity correction; for Spearman correlation coefficients, 95% CIs were derived using Fisher’s *z* transformation. Multiple imputation sensitivity analyses were pre-specified for primary BU endpoints and are reported in the companion primary manuscript^15^; the QoL outcomes herein were analyzed using the primary NRI/BOCF approach.

No adjustment for multiple comparisons was made given the exploratory nature of these analyses; results should be interpreted as hypothesis-generating.

All statistical analyses were generally performed using SAS version 9.4 or higher.

### Ethical considerations

The study was conducted in accordance with the Declaration of Helsinki, International Conference on Harmonization, Good Clinical Practice guidelines, and Council for International Organizations of Medical Sciences International Ethical Guidelines, and applicable laws and regulations as needed. The study protocol, protocol amendments, and other relevant documents were approved by independent review board or ethics committee for each study site. All patients provided written informed consent before study participation.

## Results

### Patient demographics and baseline characteristics

The analysis population included 172 patients with UC who received at least 1 dose of mirikizumab. Of these, 155 (90.1%) completed the week 12 assessment, and 134 (77.9%) completed the study through week 28. Of 172 patients, 38 (22.1%) discontinued treatment through week 28, primarily due to lack of efficacy (21 [12.2%]) and adverse events (6 [3.5%]); only 1 patient was lost to follow-up. Detailed disposition has been reported previously.^15^ The mean age of the patients was 41.1 (SD 14.4) years. The majority were male (58.7%) and White (93.2%) (Table 1). Table 1 summarizes baseline BU and QoL measures. Comprehensive baseline characteristics have been described in detail elsewhere.^15^

**Table 1:** Patient demographics and baseline characteristics.

	Mirikizumab 300 mg IV/200 mg SC *N* = 172
**Age (years), mean (SD)**	41.1 (14.4)
**Male, *n* (%)**	101 (58.7)
**BMI (kg/m^2^), mean (SD)**	25.9 (5.9)
**Race, *n* (%)^a^**	
** White**	150 (93.2)
** Black or African American**	5 (3.1)
** Asian**	4 (2.5)
** Other^a^**	2 (1.2)
**Duration of UC (years), mean (SD)^b^**	8.0 (8.2)
**Disease location, *n* (%)**	
** Ulcerative proctitis**	4 (2.3)
** Left-sided UC (distal UC)**	90 (52.3)
** Extensive ulcerative colitis/pancolitis**	78 (45.3)
**BU measures**	
** UNRS score, mean (SD)**	6.9 (1.6)
** SDT^c^, *n* (%)**	4.7 (11.5)
** <1 minute**	34 (19.8)
** ≥1-<2 minutes**	59 (34.3)
** ≥2-<5 minutes**	57 (33.1)
** ≥5-<15 minutes**	15 (8.7)
** ≥15 minutes or no urgency**	7 (4.1)
** BU frequency, times/day**	6.9 (3.7)
**IBDQ total score, mean (SD)**	106.2 (27.9)
** Bowel symptoms**	33.7 (8.3)
** Systemic symptoms**	14.9 (5.2)
** Emotional function**	41.0 (11.5)
** Social function**	16.5 (7.4)
**Baseline WPAI: UC score, mean (SD)**	
** Absenteeism**	29.8 (39.0)
** Presenteeism**	53.0 (21.3)
** Activity impairment**	60.7 (21.8)
** Work productivity loss**	57.5 (23.6)
**Baseline WIS score, mean (SD)**	
** Solid stool**	1.4 (1.4)
** Liquid stool**	2.6 (1.3)
** Gas**	2.9 (1.3)
** Use pads**	1.2 (1.6)
** Lifestyle altered**	2.9 (1.3)
**APU for BU^d^, *n* (%)**	
** Never**	75 (44.9)
** Sometimes**	21 (12.6)
** Rarely**	21 (12.6)
** Usually**	23 (13.8)
** Always**	27 (16.2)
**Baseline SAA^e^**—**frequency of avoidance, mean (SD)**	1.3 (0.9)
** Did not avoid**	30 (21.1)
** Avoided sometimes**	57 (40.1)
** Avoided most of the times**	41 (28.9)
** Avoided always**	14 (9.9)
**Baseline GAD-7 score, mean (SD)**	10.1 (5.5)

Baseline is defined as the last non-missing value prior to the first dose of study intervention.

a 1 (0.6%) American Indian/Alaska Native and 1 (0.6%) Multiple (N = 161).

b Length of the interval from the date of UC diagnosis to the date of informed consent.

c SDT was analyzed by the shortest weekly SDT, taking the shortest response (ie, minimum value) available in the past 7 days, where no urgency was considered the largest value.

d Score indicates absorbent products used by patients due to BU.

e Patients who answered not applicable at baseline are not included in the analysis.

Abbreviations: APU, absorbent product use; BMI, body mass index; BU, bowel urgency; GAD-7, Generalized Anxiety Disorder 7; IBDQ, Inflammatory Bowel Disease Questionnaire; IV, intravenous; *N*, number of patients in the analysis population; *n*, number of patients in the specified category; SAA, sexual activity avoidance; SC, subcutaneous; SD, standard deviation; SDT, shortest weekly stool deferral time; UC, ulcerative colitis; UNRS, Urgency Numeric Rating Scale; WIS, Wexner Incontinence Score; WPAI: UC, Work Productivity and Activity Impairment: Ulcerative Colitis.

### Improvements in BU measures

Briefly, all BU measures showed marked improvement over the 28-week treatment period. The mean UNRS score improvement from baseline was 3.2 (SD 2.5) points at week 12 and 3.6 (SD 3.1) points at week 28 (Figure S1). Clinically meaningful improvement in BU severity was achieved by 92 (53.5%) patients at week 12 (*n* = 172) and by 100 (58.1%) patients at week 28 (*n* = 172). BU remission was reported in 38 (22.1%) patients at week 12 (*n* = 172), and in 65 (37.8%) patients at week 28 (*n* = 172). The mean BU frequency decreased by 3.8 episodes/day at week 12, with a sustained reduction maintained through week 28. SDT values improved from baseline at weeks 12 and 28. At baseline (*n* = 172), 19.8% patients had an SDT of <1 minute, while 4.1% had an SDT of ≥15 minutes or reported no urgency. By week 12 (*n* = 172), these proportions improved to 8.7% and 15.7%, respectively. At week 28 (*n* = 172), the distribution further shifted, with 8.1% of patients in the <1-minute category and 29.7% in the ≥15 minutes or no urgency category.

Detailed findings on BU outcomes have been reported previously.^15^

### Inflammatory Bowel Disease Questionnaire

The total IBDQ score at baseline was 106.2 (SD 27.9). IBDQ total score improved by 51.2 points (SD 37.5; 95% CI: 45.4, 57.1) at week 12 and by 56.4 points (SD 45.8; 95% CI: 49.2, 63.5) at week 28. Patients also experienced consistent improvements across all 4 IBDQ domains at both follow-up time points (Figure 1A).

**Figure 1:**
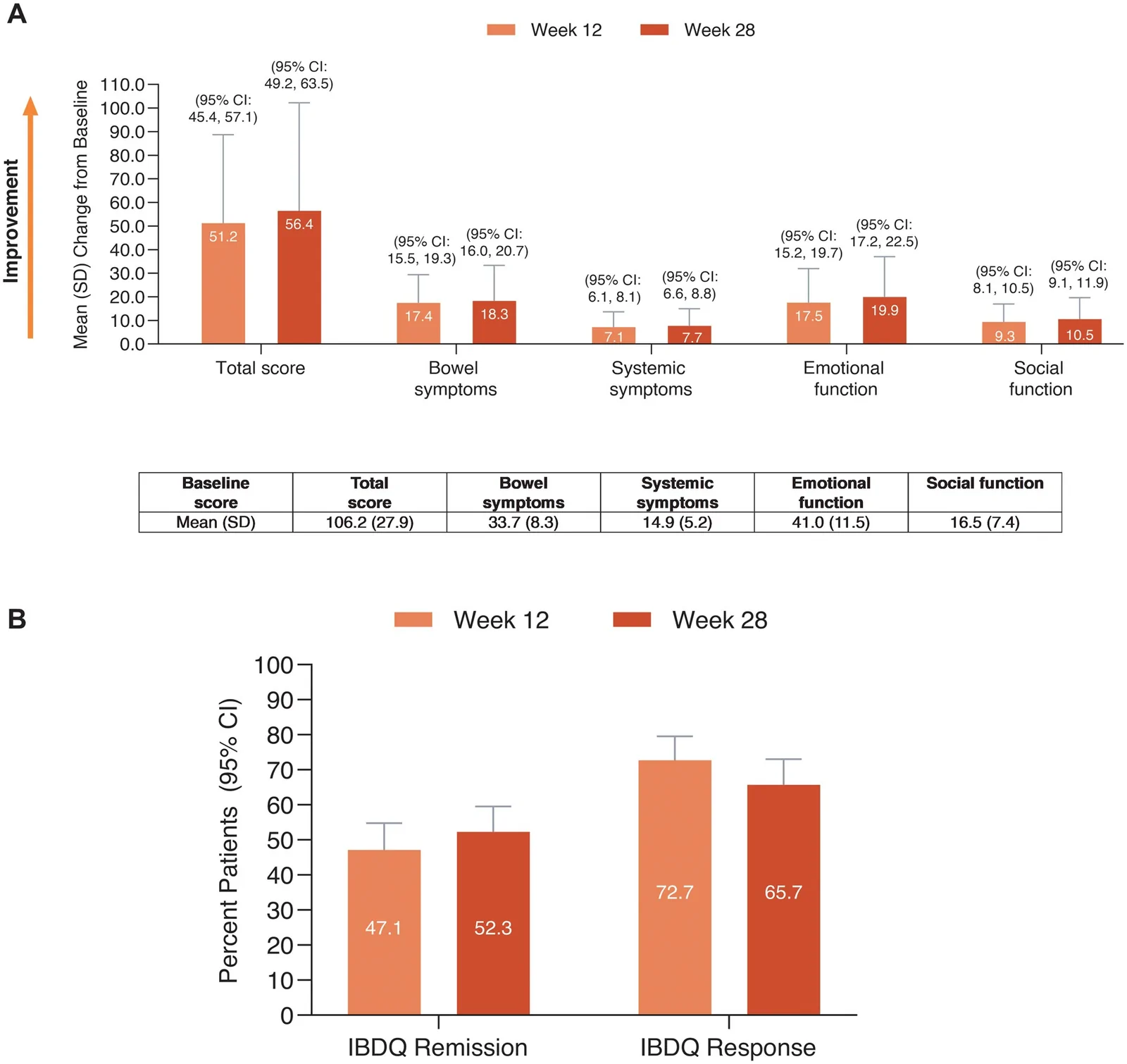
Inflammatory Bowel Disease Questionnaire (IBDQ) outcomes: (A) Mean (SD) change from baseline in total and domain scores at weeks 12 and 28; (B) Proportion of patients achieving IBDQ remission^a^ and response^b^ at weeks 12 and 28. IBDQ total score: *N* at baseline = 160; *N* at week 12 = 169; *N* at week 28 = 165; BOCF. IBDQ remission and response: *N* = 172. ^a^IBDQ remission: total IBDQ score of ≥170. ^b^IBDQ response: improvement of ≥16 points in the total IBDQ score. For IBDQ response and remission, 95% CIs for proportions were calculated using (asymptotic method without continuity correction [that is, normal approximation to the binomial distribution method]). BOCF, baseline observation carried forward; CI, confidence interval; IBDQ, Inflammatory Bowel Disease Questionnaire; *N*, number of patients in the analysis population; SD, standard deviation.

At week 12, 47.1% (*n* = 81/172) of patients achieved IBDQ remission, increasing to 52.3% (*n* = 90/172) by week 28 as continued treatment enabled additional patients to reach the absolute IBDQ threshold (≥170). IBDQ response (≥16-point improvement from baseline) was achieved by 72.7% (*n* = 125/172) of patients at week 12 and 65.7% (*n* = 113/172) at week 28. The modest decline in response rate at week 28 reflects NRI: patients who discontinued between weeks 12 and 28 were classified as non-responders at week 28, and because a greater proportion had achieved response than remission at week 12, the response rate was disproportionately affected by discontinuations (Figure 1B).

### Work Productivity and Activity Impairment: Ulcerative Colitis

Baseline scores (Figure 2) ranged from 29.8% for absenteeism to 60.7% for activity impairment. At week 12, all 4 WPAI: UC domains showed notable improvements, with mean reductions in absenteeism score by 20.0%, presenteeism score by 23.4%, overall work productivity score by 25.8%, and activity impairment score by 28.8%. By week 28, these improvements were either sustained or exhibited marginal enhancement across most domains (Figure 2).

**Figure 2:**
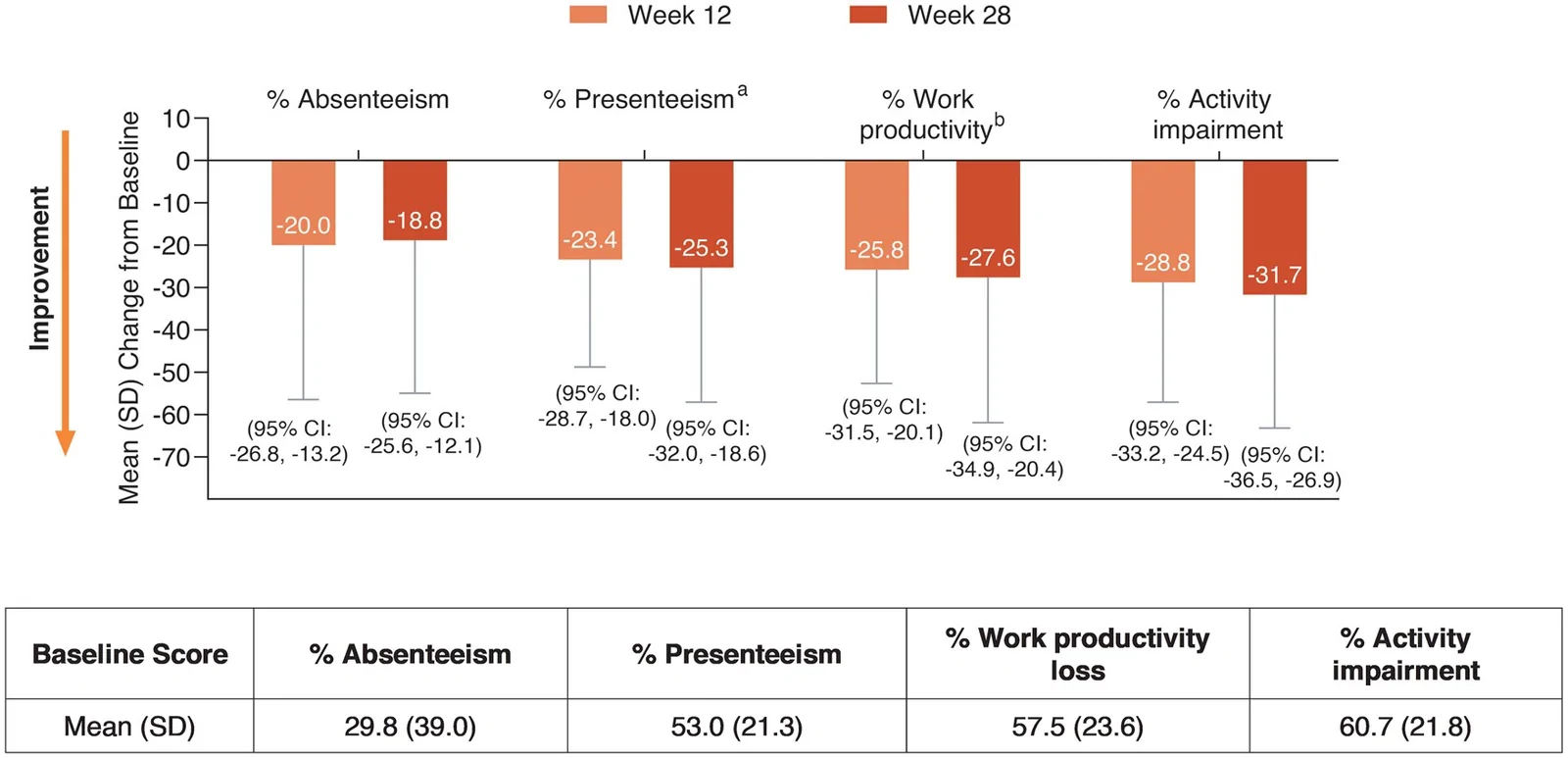
Mean (SD) change from baseline in work productivity and activity impairment at weeks 12 and 28. Baseline, *N* = 165; week 12, *N* = 170; week 28, *N* = 167; BOCF. ^a^Reduced productivity while at work. ^b^Overall work productivity score is an aggregate of absenteeism and presenteeism. Activity impairment score was analyzed for all patients; other components were for participants with employment only. BOCF, baseline observation carried forward; CI, confidence interval; *N*, number of patients in the analysis population; SD, standard deviation.

### Absorbent product use for BU

At baseline, 55.1% (*n* = 92/167) of patients reported APU for BU (ranging from “sometimes” to “always”), which decreased to 34.7% (*n* = 59/170) at week 12 and 29.6% (*n* = 50/169) at week 28. As shown in Figure 3, 44.9% (*n* = 75/167) at baseline reported no APU, which increased to 70.4% (*n* = 119/169) at week 28.

**Figure 3:**
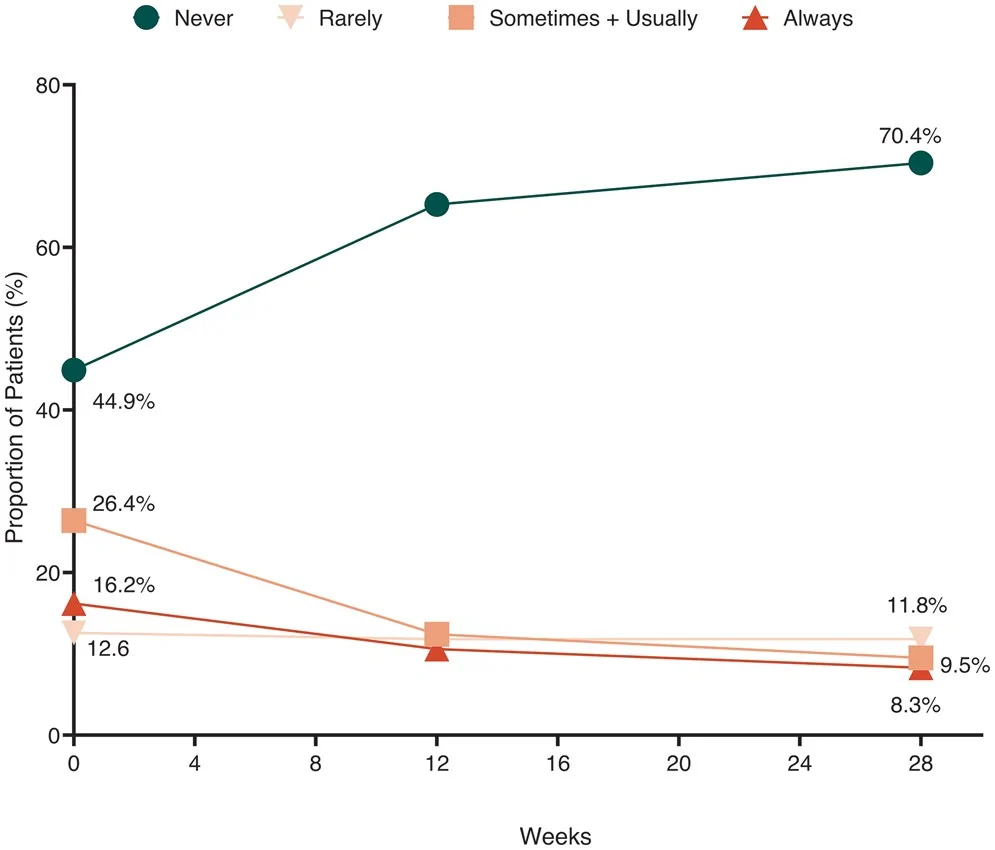
Absorbent product use for bowel urgency: Patient distribution by frequency of absorbent product use. BOCF, baseline observation carried forward; BU, bowel urgency; *N*, number of patients in the analysis population.

### Sexual activity avoidance due to UC symptoms

Proportion of patients reporting no SAA due to UC symptoms increased from 21.1% (*n* = 30/142) at baseline to 45.8% (*n* = 65/142) at week 12, with corresponding reductions in higher avoidance categories. This trend continued through week 28, with 51.4% (*n* = 73/142) reporting no SAA due to UC symptoms (Figure 4).

**Figure 4:**
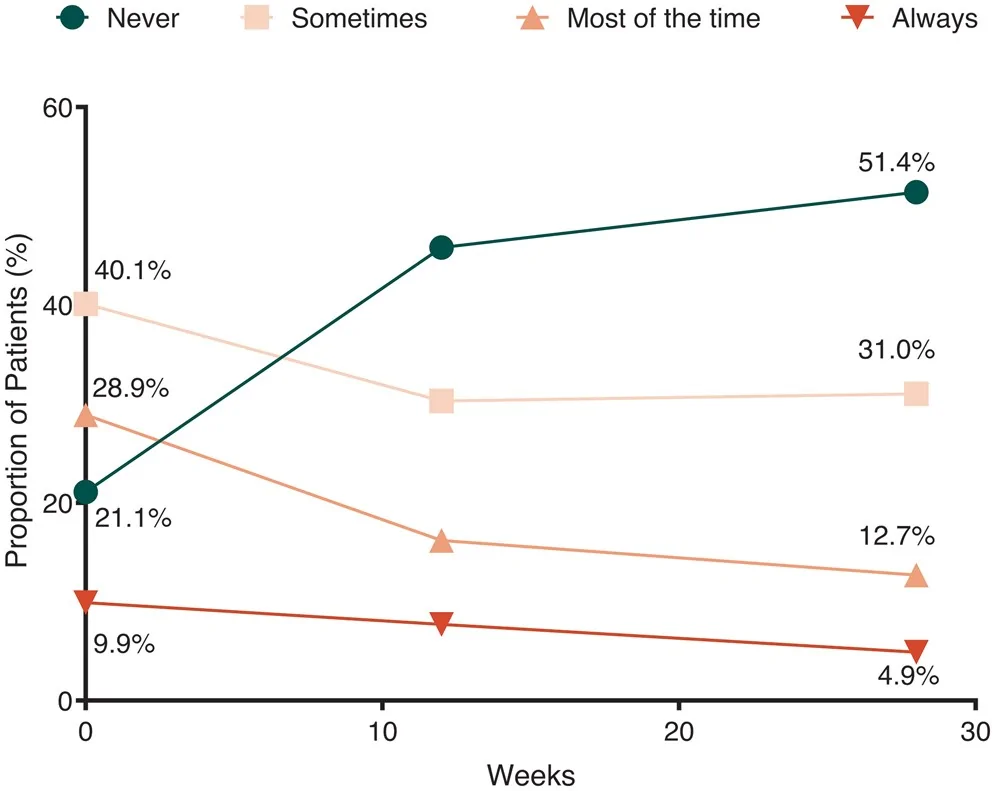
Patient responses to sexual activity avoidance question at baseline, weeks 12 and 28. ^a,b a^Baseline, *N* = 142; Week 12, *N* = 142; Week 28, *N* = 142. BOCF. ^b^Patients who answered “N/A” or no response at sexual activity avoidance question 1 at baseline were excluded from the analysis. BOCF, baseline observation carried forward; *N*, number of patients in the analysis population.

Common reasons for SAA at baseline included BU (32.0%), tiredness/fatigue (31.4%), abdominal pain (25.0%), decreased sexual desire (22.1%), and blood in stool (15.1%). By week 28, these concerns had declined numerically to 14.5%, 7.6%, 8.1%, 11.6%, and 1.7%, respectively. Several less frequently reported reasons (eg, fear of BU-related accidents and increased stool frequency) also showed notable reductions. However, some concerns, such as feeling physically unattractive, difficulty reaching orgasm/climax, and self-consciousness about cleanliness, remained relatively stable by week 28 (Table S2).

### Wexner Incontinence Score

Fecal incontinence, assessed using the WIS, showed notable improvement across all 5 domains from baseline to week 12, with these gains sustained through week 28 (Figure 5A).

**Figure 5:**
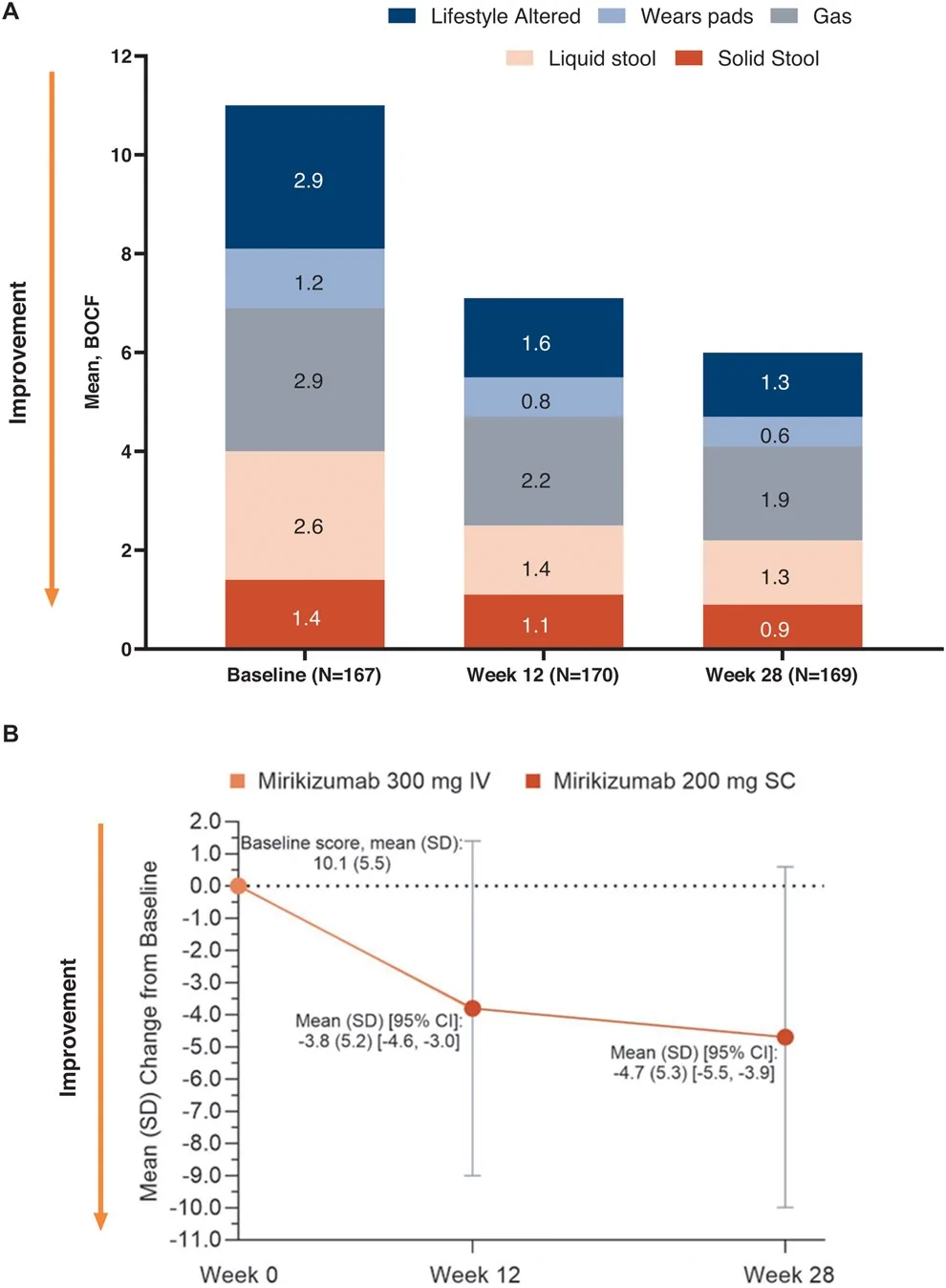
Change from baseline in (A) incontinence-related functional impact (Wexner Incontinence Score^a^) and (B) anxiety symptom severity (Generalized Anxiety Disorder-7^b^). ^a^Baseline, *N* = 167; week 12, *N* = 170; week 28, *N* = 169; BOCF. ^b^Baseline, *N* = 165; week 12, *N* = 170; week 28, *N* = 167; BOCF. BOCF, baseline observation carried forward; CI, confidence interval; IV, intravenous; N, number of patients in the analysis population; SC, subcutaneous; SD, standard deviation.

### General Anxiety Disorder-7

Anxiety symptoms, assessed using the GAD-7 scale, declined from a baseline mean of 10.1 (SD 5.5) by 3.8 (SD 5.2) at week 12 and by 4.7 (SD 5.3) at week 28 (Figure 5B). A baseline GAD-7 score ≥10 corresponds to the established threshold for moderate anxiety,^31^ and highlights the burden at study entry and improvements that are likely to be meaningful from a patient perspective.

### Correlations between BU measures with PROs

The BU measures (BU severity [UNRS], BU frequency, and SDT) showed very weak to weak correlations with most other PROs (SAA, APU, WIS, and GAD-7) at baseline, with predominantly weak correlations maintained through weeks 12 and 28 (Table 2).

**Table 2:** Correlations between bowel urgency measures and patient-reported outcomes at baseline, week 12, and week 28.

IBDQ					
	IBDQ total score	IBDQ subscores			
		Bowel symptoms	Systemic symptoms	Emotional function	Social function
**Baseline: analysis value^a^; Spearman correlation coefficient (95% CI)**					
**UNRS**	−0.28 (−0.42, −0.13)	−0.27 (−0.41, −0.12)	−0.30 (−0.43, −0.15)	−0.22 (−0.36, −0.06)	−0.26 (−0.40, −0.11)
**BUF**	−0.36 (−0.49, −0.22)	−0.38 (−0.50, −0.23)	−0.37 (−0.49, −0.22)	−0.19 (−0.34, −0.04)	−0.43 (−0.55, −0.29)
**SDT**	0.14 (−0.01, 0.29)	0.16 (0.00, 0.31)	0.14 (−0.02, 0.29)	0.09 (−0.07, 0.24)	0.14 (−0.01, 0.29)
**Week 12: analysis value^a^; Spearman correlation coefficient (95% CI)**					
**UNRS**	−0.55 (−0.65, −0.42)	−0.48 (−0.60, −0.34)	−0.50 (−0.61, −0.37)	−0.51 (−0.62, −0.38)	−0.49 (−0.60, −0.36)
**BUF**	−0.60 (−0.70, −0.49)	−0.57 (−0.67, −0.45)	−0.57 (−0.67, −0.45)	−0.51 (−0.62, −0.38)	−0.59 (−0.68, −0.47)
**SDT**	0.37 (0.20, 0.51)	0.35 (0.19, 0.49)	0.35 (0.19, 0.49)	0.30 (0.14, 0.45)	0.36 (0.20, 0.50)
**Week 28: analysis value^a^; Spearman correlation coefficient (95% CI)**					
**UNRS**	−0.44 (−0.57, −0.28)	−0.43 (−0.57, −0.27)	−0.40 (−0.54, −0.23)	−0.39 (−0.53, −0.23)	−0.37 (−0.51, −0.20)
**BUF**	−0.51 (−0.63, −0.36)	−0.47 (−0.60, −0.32)	−0.46 (−0.59, −0.30)	−0.46 (−0.59, −0.30)	−0.50 (−0.62, −0.35)
**SDT**	0.20 (0.00, 0.39)	0.28 (0.08, 0.46)	0.18 (−0.03, 0.38)	0.11 (−0.10, 0.31)	0.22 (0.01, 0.41)

WPAI: UC				
	Work productivity loss	Absenteeism	Presenteeism	Activity impairment
**Baseline: analysis value^a^; Spearman correlation coefficient (95% CI)**				
**UNRS**	0.21 (0.00, 0.40)	−0.01 (−0.19, 0.18)	0.29 (0.09, 0.47)	0.28 (0.13, 0.42)
**BUF**	0.20 (−0.01, 0.39)	0.09 (−0.09, 0.27)	0.24 (0.03, 0.43)	0.27 (0.12, 0.40)
**SDT**	−0.29 (−0.47, −0.09)	−0.05 (−0.23, 0.14)	−0.30 (−0.48, −0.09)	−0.10 (−0.25, 0.05)
**Week 12: analysis value^a^; Spearman correlation coefficient (95% CI)**				
**UNRS**	0.40 (0.22, 0.56)	0.28 (0.10, 0.45)	0.40 (0.21, 0.55)	0.53 (0.40, 0.64)
**BUF**	0.50 (0.34, 0.64)	0.27 (0.08, 0.44)	0.50 (0.34, 0.64)	0.59 (0.48, 0.69)
**SDT**	−0.34 (−0.51, −0.13)	−0.21 (−0.39, 0.00)	−0.35 (−0.52, −0.14)	−0.29 (−0.44, −0.13)
**Week 28: analysis value^a^; Spearman correlation coefficient (95% CI)**				
**UNRS**	0.21 (−0.01, 0.41)	0.05 (−0.16, 0.25)	0.23 (0.01, 0.42)	0.49 (0.34, 0.61)
**BUF**	0.29 (0.07, 0.47)	0.07 (−0.14, 0.28)	0.30 (0.09, 0.48)	0.56 (0.42, 0.67)
**SDT**	−0.08 (−0.33, 0.18)	−0.11 (−0.34, 0.14)	−0.09 (−0.34, 0.17)	−0.24 (−0.42, −0.04)

PRO measures				
	WIS	APU for BU	GAD-7	SAA due to UC
**Baseline: analysis value^a^; Spearman correlation coefficient (95% CI)**				
**UNRS**	0.27 (0.12, 0.40)	0.29 (0.14, 0.42)	0.18 (0.02, 0.32)	0.15 (−0.01, 0.31)
**BUF**	0.27 (0.13, 0.41)	0.36 (0.22, 0.48)	0.12 (−0.03, 0.27)	0.16 (0.00, 0.32)
**SDT**	−0.26 (−0.39, −0.11)	−0.15 (−0.29, 0.01)	−0.08 (−0.23, 0.07)	−0.20 (−0.35, −0.03)
**Week 12: analysis value^a^; Spearman correlation coefficient (95% CI)**				
**UNRS**	0.40 (0.25, 0.52)	0.37 (0.23, 0.50)	0.36 (0.21, 0.49)	0.35 (0.18, 0.49)
**BUF**	0.47 (0.34, 0.58)	0.45 (0.31, 0.57)	0.35 (0.21, 0.49)	0.44 (0.29, 0.57)
**SDT**	−0.33 (−0.47, −0.17)	−0.41 (−0.54, −0.26)	−0.20 (−0.36, −0.03)	−0.23 (−0.40, −0.03)
**Week 28: analysis value^a^; Spearman correlation coefficient (95% CI)**				
**UNRS**	0.31 (0.14, 0.46)	0.23 (0.06, 0.39)	0.22 (0.05, 0.38)	0.20 (0.01, 0.38)
**BUF**	0.40 (0.24, 0.54)	0.36 (0.19, 0.50)	0.32 (0.15, 0.47)	0.39 (0.21, 0.54)
**SDT**	−0.17 (−0.36, 0.03)	−0.31 (−0.48, −0.11)	−0.09 (−0.28, 0.12)	−0.05 (−0.28, 0.18)

**Level of correlation**	0.00 to 0.09 No/negligible	0.10 to 0.29 Very Weak	0.30 to 0.49 Weak	0.50 to 0.69 Moderate	0.70 to 0.89 Strong	≥0.90 Very Strong

a Only observed data were used for correlation. Fisher’s Z transformation was used to calculate 95% CIs.

Abbreviations: APU, absorbent product use; BU, bowel urgency; BUF, bowel urgency frequency; CI, confidence interval; GAD-7, Generalized Anxiety Disorder 7; IBDQ, Inflammatory Bowel Disease Questionnaire; PRO, patient-reported outcome; SAA, sexual activity avoidance; SDT, shortest weekly stool deferral time; UC, ulcerative colitis; UNRS, Urgency Numeric Rating Scale; WIS, Wexner Incontinence Score; WPAI: UC, Work Productivity and Activity Impairment: Ulcerative Colitis.

At baseline, BU measures showed weak correlations with IBDQ scores. By week 12, BU severity and frequency were moderately correlated with several IBDQ domains, with BU frequency maintaining moderate correlations with the total and social function scores at week 28 (Table 2).

BU measures showed weak correlations with WPAI: UC components, except at week 12, where BU severity had moderate correlations with activity impairment while BU frequency had moderate correlations with activity impairment, work productivity loss, and presenteeism (Table 2).

In contrast, SMDs showed large associations between BU CMI and IBDQ, and medium-to-large associations between BU remission and IBDQ. Changes in BU severity and BU frequency were largely associated with both IBDQ response and remission, whereas change in SDT showed low to medium associations with these outcomes. Associations with WPAI: UC outcomes were generally medium, with the largest associations observed between BU remission or CMI and activity impairment (Table 3).

**Table 3:** Associations at weeks 12 and 28 between: (A) bowel urgency improvement and Inflammatory Bowel Disease Questionnaire (IBDQ) and work productivity, and (B) IBDQ improvement and bowel urgency measures.

IBDQ					
	IBDQ total score	IBDQ Subscores			
		Bowel symptoms	Systemic symptoms	Emotional function	Social function
**Week 12: analysis value^a^; SMD (95% CI)**					
**BU remission^b^**	0.96 (0.61, 1.30)	0.88 (0.53, 1.22)	0.91 (0.57, 1.26)	0.86 (0.51, 1.20)	0.87 (0.53, 1.21)
**BU CMI^c^**	1.05 (0.70, 1.40)	1.01 (0.66, 1.36)	1.00 (0.65, 1.34)	0.94 (0.60, 1.29)	0.86 (0.51, 1.20)
**Week 28: analysis value^a^; SMD (95% CI)**					
**BU remission^b^**	0.80 (0.42, 1.17)	0.68 (0.31, 1.05)	0.83 (0.46, 1.21)	0.76 (0.38, 1.13)	0.64 (0.27, 1.01)
**BU CMI^c^**	1.08 (0.70, 1.47)	1.00 (0.62, 1.39)	0.82 (0.45, 1.20)	1.03 (0.65, 1.41)	1.04 (0.66, 1.43)

WPAI: UC				
	Work productivity loss score	Absenteeism score	Presenteeism score	Activity Impairment score
**Week 12: analysis value^a^; SMD (95% CI)**				
**BU remission^b^**	−0.71 (−1.12, −0.29)	−0.46 (−0.84, −0.07)	−0.67 (−1.09, −0.26)	−1.02 (−1.37, −0.68)
**BU CMI^c^**	−0.57 (−0.98, −0.16)	−0.29 (−0.67, 0.09)	−0.57 (−0.98, −0.16)	−0.92 (−1.25, −0.58)
**Week 28: analysis value^a^; SMD (95% CI)**				
**BU remission^b^**	−0.47 (−0.91, −0.03)	−0.26 (−0.68, 0.16)	−0.50 (−0.95, −0.06)	−0.82 (−1.19, −0.45)
**BU CMI^c^**	−0.64 (−1.08, −0.19)	−0.54 (−0.96, −0.11)	−0.65 (−1.09, −0.20)	−1.04 (−1.41, −0.66)

Association between BU measures and IBDQ endpoints			
	UNRS (BU severity)	BU frequency	SDT
**Week 12: analysis value^a^; SMD (95% CI)**			
**IBDQ remission^d^**	−1.03 (−1.38, −0.68)	−1.19 (−1.54, −0.83)	0.35 (0.01, 0.67)
**IBDQ response^e^**	−1.26 (−1.63, −0.89)	−0.97 (−1.32, −0.61)	0.30 (−0.04, 0.64)
**Week 28: analysis value^a^; SMD (95% CI)**			
**IBDQ remission^d^**	−0.82 (−1.20, −0.44)	−0.98 (−1.36, −0.60)	0.55 (0.18, 0.92)
**IBDQ response^e^**	−0.89 (−1.28, −0.51)	−1.06 (−1.45, −0.66)	0.09 (−0.28, 0.46)

**Association by SMD value (±)^54^**	0.20 to <0.50 Small	0.50 to <0.80 Medium	≥0.80 Large

a Only observed data was used for association. Cohen’s *d* is used to calculate SMD and corresponding confidence intervals.

b BU remission: rounded UNRS score ≤1.0.

c BU CMI: A ≥ 3-point reduction from baseline in the UNRS score.

d IBDQ remission: total IBDQ score of ≥170.

e IBDQ response: improvement of ≥16 points in the total IBDQ score.

Abbreviations: BU, bowel urgency; CI, confidence interval; CMI, clinically meaningful improvement; IBDQ, Inflammatory Bowel Disease Questionnaire; SDT, shortest weekly stool deferral time; SMD, standardized mean difference; UC, ulcerative colitis; UNRS, Urgency Numeric Rating Scale; WPAI: UC, Work Productivity and Activity Impairment: Ulcerative Colitis.

## Discussion

LUCENT-URGE, a phase 3b study, is the first to exclusively enroll patients experiencing BU at baseline, enabling a focused evaluation of various aspects of BU, beyond severity, affecting QoL in patients with moderately-to-severely active UC.^15^ BU correlates with conventional clinical and endoscopic outcomes but does not fully reflect inflammatory activity and may persist despite improvement in these measures.^15^ This highlights its value as a complementary, patient-relevant outcome capturing a distinct aspect of UC burden not fully assessed by traditional endpoints.

Aligning with findings from LUCENT-1 and LUCENT-2 trials, treatment with mirikizumab in this study was associated with clinically meaningful improvements in BU severity as well as frequency and SDT as early as week 12, with sustained or enhanced benefits observed through week 28. Notably, over half of the patients achieved CMI in BU severity, and more than one-third attained BU remission by week 28.

BU is a multidimensional symptom influenced by deferral time, frequency, contextual factors (eg, bathroom accessibility), adaptive behaviors, and the presence of fecal incontinence.^12^ Despite its substantial impact, BU remains underrepresented in clinical trials and is often overlooked in routine clinical practice.^14^ The LUCENT-URGE study addressed this critical gap by employing BU-specific measures: BU severity (UNRS), BU frequency, and SDT.^15^ These measures provide a comprehensive assessment of the multidimensional nature of BU and offer deeper insights into symptom burden and treatment response. Moreover, the use of both validated (UNRS) and novel BU metrics (BU frequency and SDT) in this study establishes a robust framework for future studies aimed at capturing the full spectrum of BU-related burden in UC.

A meta-analysis on QoL in IBD emphasized that while generic HRQoL measures allow cross-population comparisons, they may underestimate IBD’s burden. Disease-specific instruments, such as IBDQ, are therefore more appropriate for IBD-focused research.^33–36^ In the current study, improvements in BU were paralleled by substantial gains in HRQoL. By week 28, 65.7% of mirikizumab-treated patients achieved an IBDQ response, and 52.3% reached IBDQ remission. These findings align with recent data demonstrating strong associations between IBDQ scores, treatment efficacy, and clinical improvement.^37^ UC symptoms are a major driver of work-related disability, increasing both absenteeism and presenteeism, thereby raising unemployment and overall economic burden.^38–40^ Notably, in a recent survey, over 40% of physicians (262/654) identified improving patients’ ability to work or attend school as one of the top 3 HRQoL goals in IBD care.^41^ In this study, treatment with mirikizumab was associated with sustained and progressive improvements across all 4 WPAI: UC domains through week 28. The greatest benefit was observed in reduced activity impairment, highlighting substantial gains in patients’ daily functioning. These improvements in disease-specific QoL (IBDQ and WPAI: UC) align with the long-term improvements reported in the LUCENT-1 and LUCENT-2 trials, though caution is warranted when comparing across different study durations.^23^

BU and its associated fear of fecal incontinence are strongly linked to increased risks of social impairment, depression, anxiety, and broader psychological and physical burdens, including impact on sexual health, in patients with UC. These wide-ranging impacts underscore the importance of evaluating outcomes that go beyond traditional PROs.^42–44^ In this study, novel PROs were assessed, including APU for BU, SAA due to UC symptoms, fecal incontinence (measured by WIS), and anxiety (GAD-7). These domains reflect critical yet often underrepresented aspects of patient experience, specifically continence, intimacy, and mental health. Notably, SAA assessed the participants’ avoidance of sexual activity, including masturbation, allowing the inclusion of all participants, even those who do not have an intimate partner.^45^ Overall, incorporating these PROs enables a more comprehensive understanding of disease burden, supports individualized care, and aligns with patient-centered regulatory priorities. Improvements in these domains were aligned with clinical progress, underscoring their value in assessing treatment efficacy. By week 28, a substantially greater proportion of patients reported reduced or discontinued absorbent products and reduced SAA, fecal incontinence, indicating reduced symptom severity and improved bowel control. Improvements in the IBDQ Emotional Function domain and anxiety highlight meaningful benefits in psychological well-being, a key dimension of disease burden in IBD. The mean reduction in GAD-7 scores at week 28 (4.7 points) exceeded published lower-threshold estimates of the minimal clinically important difference (MCID, 3.3–4.0 points),^46^^,^^47^ indicating a clinically meaningful improvement in anxiety symptoms. These consistent and sustained improvements underscore the multidimensional benefits of mirikizumab in alleviating physical symptoms, restoring daily functioning, and enhancing confidence and emotional well-being in patients with UC.

Overall, correlations between BU measures and PROs such as SAA, APU, WIS, and GAD-7 were weak from baseline through week 28, indicating a potential opportunity to explore whether alternative or complementary instruments might provide additional insights into BU-related measures. However, this requires further investigation as other factors, such as differences in patient-reported symptoms perception, variability in disease duration, or differences in treatment adherence could contribute to the variability in the observed scores.^48–50^ The weak correlation between BU and GAD-7 is consistent with the multifactorial nature of anxiety in UC, which encompasses contributors beyond gastrointestinal symptom severity, including chronic disease burden, prior corticosteroid exposure, uncertainty related to relapse, social and occupational limitations, and comorbid mood disorders.

The observed correlations between BU severity, frequency, and SDT, and various PROs offer valuable insights into how symptom severity affects patients’ QoL and functioning. Correlations between BU measures and the IBDQ total/domain scores varied over time, with a numerical peak at week 12 and attenuation by week 28. At week 12, BU severity and frequency showed moderate correlations with several IBDQ domains and WPAI: UC activity impairment, suggesting that symptom improvements are linked to better daily functioning and perceived health status. Notably, BU frequency maintained consistent correlations with the IBDQ total score, the social functioning domain, and activity impairment through week 28. These findings support the importance of symptom relief in restoring social functioning and suggest that BU frequency may be a sensitive and responsive indicator of QoL and functional outcomes.

BU remission showed a large and CMI showed medium-to-large association with IBDQ. Both BU remission and CMI had large associations with reduced activity impairment and showed small-to-medium correlations with other WPAI: UC domains. Together, these findings highlight the tangible benefits of symptom resolution on daily functioning and reinforce the clinical relevance of achieving remission or meaningful improvement. These were further supported by large associations between BU measures and IBDQ endpoints, reinforcing the multidimensional impact of symptom control. These findings are consistent with earlier evidence highlighting the significant impact of BU on QoL in patients with moderately-to-severely active UC.^17^^,^^18^^,^^22^

Collectively, these data provide novel insights showing that improvements in BU measures are directionally aligned with improvements in QoL. Alleviating BU not only reduces symptom burden but also enhances patients’ ability to resume normal daily activities, including work and social participation. These findings emphasize the clinical value of assessing and managing BU as a part of comprehensive UC care. BU CMI and remission represent patient-centered outcomes that may complement established clinical and endoscopic treatment goals. Because BU may reflect mechanisms beyond mucosal inflammation, including altered rectal function and reduced rectal compliance,^12^^,^^51^^,^^52^ continued assessment of BU may help capture residual patient burden even when inflammatory markers or endoscopic outcomes improve. A study has shown increased anal sphincter “fatigability” in patients with UC who experience urgency; importantly, this fatigability is independent of local inflammation.^53^

A key strength of this study is its novel design. To our knowledge, it is the first study to specifically include patients with BU and to explore additional aspects of BU beyond severity affecting QoL in patients with UC. The study employed a comprehensive set of validated disease-specific and generic HRQoL instruments, enabling a multidimensional assessment of treatment outcomes.

However, several limitations should be acknowledged. As an open-label, single-arm study, LUCENT-URGE lacks a comparator arm, limiting causal inference.^15^ Nonetheless, the consistency of these findings with prior placebo-controlled data from LUCENT-1 and -2 enhances their interpretability and supports the robustness of the observed effects. Clinical and endoscopic outcomes were collected and reported in the primary LUCENT-URGE study^15^; however, these were not the focus of the present QoL and correlation analyses. Given that the focus of this manuscript is on QoL endpoints, data on the presence and severity of rectal inflammation at baseline and at the end of treatment are not presented here. Segment-specific histologic assessments of rectal inflammation will be reported in future analysis.

The inclusion of multiple PROs increases the potential for multiplicity; however, the concordance of results across diverse measures strengthens the conclusions. Although the relatively short duration of 28 weeks limits the ability to assess long-term durability, the sustained improvements observed within this timeframe are encouraging and consistent with previously published data from the LUCENT clinical trial program, which includes maintenance of outcomes extending up to 1 year.

Furthermore, although the inclusion of novel BU-specific metrics provides valuable insights, further validation in independent cohorts is warranted to confirm their generalizability and sensitivity. In addition, the predominantly White study population may limit the applicability of these findings to more racially and ethnically diverse populations. Lastly, the reliance on self-reported data, though necessary for capturing patient experience, may also introduce reporting bias. Future studies involving larger, more diverse populations should, where feasible, incorporate objective measures with PROs to strengthen assessment of treatment impact and reduce potential reporting bias.

## Conclusion

Mirikizumab treatment was associated with comprehensive and sustained improvements across multiple dimensions of BU, including severity, frequency, and SDT, with clinically meaningful benefits evident by the primary assessment at week 12 and further enhanced by week 28 in patients with UC. These improvements were accompanied by reduction in anxiety, enhanced work productivity, and meaningful improvements or remission over time in multiple validated QoL measures. Importantly, mirikizumab treatment was also associated with a reduction or resolution of negative impacts of BU, including decreased reliance on absorbent products and a reduction in avoidance of sexual activity due to UC symptoms. These findings underscore the importance of managing key patient-centric symptoms, including BU, as part of a comprehensive and holistic UC management strategy, in addition to achievement of clinical improvement and resolution of mucosal inflammation, and highlight the contribution of mirikizumab in improving critical, yet previously under-addressed dimensions of patients’ QoL.

## Supplementary Material

otag107_Supplementary_Data

## Data Availability

Individual participant data collected during the trial will be made available after anonymization. Data are available to request 6 months after the indication studied has been approved in the United States and EU and after primary publication acceptance, whichever is later. No expiration date of data requests is currently set once data are made available. Access is provided after a proposal has been approved by an independent review committee identified for this purpose and after receiving a signed data sharing agreement. Data and documents, including the study protocol, statistical analysis plan, clinical study report, blank or annotated case report forms, will be provided in a secure data sharing environment. For details on submitting a request, see the instructions provided at www.vivli.org.
