# Supplementary material for: Mirikizumab is associated with improvements in bowel urgency and quality of life in moderately-to-severely active ulcerative colitis: 28-week phase 3b results from the LUCENT-URGE trial
Source: Crohns Colitis 360. 2026 Sep 24;8(4):otag107. doi: 10.1093/crocol/otag107 (PMC13695447; doi:10.1093/crocol/otag107)
Supplement: Supplementary file 1 [file otag107_supplementary_data.zip › AMBZ QoL Manuscript_Supplementary Material_05 Oct 26.docx]

**Supplementary Material**

**Table S1: Detailed description of patient-reported QoL outcomes**

| **Patient-reported QoL measures** | |
| --- | --- |
| IBDQ^1-3^ | The IBDQ is a 32-item questionnaire that measures QoL across 4 domains: i) bowel symptoms, ii) systemic symptoms, iii) emotional function, and iv) social function. Each item is rated on a 7-point Likert scale (7=“not a problem at all”; 1=“a very severe problem”) over a recall period of last 2 weeks. The total score, calculated as the sum of the 4 domains, ranges from 32–224, with higher score indicating better QoL. |
| WPAI:UC^4,5^ | The WPAI:UC assesses the effect of UC on work productivity and regular activities over the past 7 days. This 6-item instrument generates 4 metrics: absenteeism (percentage of work time missed due to UC), presenteeism (percentage of reduced productivity while at work), work productivity loss (overall work impairment that combines absenteeism and presenteeism), and activity impairment (percentage of impairment in regular activities performed outside of work). Patients who are employed complete the absenteeism, presenteeism, and overall work impairment questions, and all patients complete the activity impairment question. Each component score ranges from 0 to 100, with higher scores indicating greater impairment and less productivity. |
| WIS^6^ | The WIS evaluates the frequency and types of fecal incontinence experienced by patients over the past 2 months. It comprises 5 items, regarding types of leakage (solid, liquid, and gas), the necessity to wear hygiene pads, and lifestyle alterations. Each item is scored based on a frequency scale ranging from “never” (0 times), “rarely” (<1/month), “sometimes” (<1/week, ≥1/month), “usually” (<1/day, ≥1/week) to “always” (≥1/day). The final score ranges from 0, indicating full continence, to 20, representing complete incontinence. |
| GAD-7^7^ | The GAD-7 is a 7-item self-reported questionnaire that assesses severity of anxiety over the past 2 weeks. For each GAD symptom, patients indicate the frequency of being bothered on a 4-point scale from “not at all” (0) to “nearly every day” (3), resulting in a total score ranging from 0 to 21. Higher scores indicate more severe GAD symptoms. |

GAD(-7), General Anxiety Disorder (7); IBDQ, Inflammatory Bowel Disease Questionnaire; QoL, quality of life; UC, ulcerative colitis; WIS, Wexner Incontinence Score; WPAI:UC, Work Productivity and Activity Impairment:Ulcerative Colitis.

1. Guyatt G, Mitchell A, Irvine EJ*, et al.* A new measure of health status for clinical trials in inflammatory bowel disease. *Gastroenterology.* 1989;96:804-10.
2. Irvine EJ, Zhou Q, Thompson AK. The short inflammatory bowel disease questionnaire: A quality of life instrument for community physicians managing inflammatory bowel disease. CCRPT investigators. Canadian Crohn's Relapse Prevention Trial. *Am J Gastroenterol.* 1996;91:1571-8.
3. Pallis AG, Mouzas IA, Vlachonikolis IG. The inflammatory bowel disease questionnaire: A review of its national validation studies. *Inflamm Bowel Dis.* 2004;10:261-9.
4. [Reilly associates] Reilly associates WPAI scoring. <http://www.reillyassociates.net/wpai_scoring.html>. Accessed Jan 15, 2026.
5. Reilly MC, Zbrozek AS, Dukes EM. The validity and reproducibility of a work productivity and activity impairment instrument. *Pharmacoeconomics.* 1993;4:353-65.
6. Jorge JM, Wexner SD. Etiology and management of fecal incontinence. *Dis Colon Rectum.* 1993;36:77-97.
7. Spitzer RL, Kroenke K, Williams JB, Lowe B. A brief measure for assessing generalized anxiety disorder: The GAD-7. *Arch Intern Med.* 2006;166:1092-7.

**Table S2.** Reasons for SAA due to UC symptoms

**Improvement**

| **Reasons for avoidance of sexual activity, n (%)** | **Baseline N=172** | **Week 12 N=172** | **Week 28 N=172** |
| --- | --- | --- | --- |
| **Patients with at least one reason** | 112 (65.1) | 62 (36.0) | 37 (21.5) |
| Tiredness/fatigue due to my UC | 54 (31.4) | 46 (26.7) | 25 (14.5) |
| Bowel urgency | 55 (32.0) | 27 (15.7) | 13 (7.6) |
| Abdominal pain due to my UC | 43 (25.0) | 25 (14.5) | 14 (8.1) |
| Decreased sexual desire due to my UC | 38 (22.1) | 34 (19.8) | 20 (11.6) |
| Blood in stool | 26 (15.1) | 9 (5.2) | 3 (1.7) |
| Fear of bowel urgency-related accidents | 18 (10.5) | 9 (5.2) | 6 (3.5) |
| Feeling physically unattractive due to my UC | 7 (4.1) | 8 (4.7) | 7 (4.1) |
| Pain around the anus due to my UC | 5 (2.9) | 7 (4.1) | 3 (1.7) |
| Difficulty reaching orgasm/climax during sexual activity | 3 (1.7) | 5 (2.9) | 4 (2.3) |
| Self-conscious about not feeling clean | 3 (1.7) | 5 (2.9) | 4 (2.3) |
| Fear of bowel movement accidents that occur without my control or any warning | 3 (1.7) | 0 | 2 (1.2) |
| Fear of fecal seepage/unnoticed leakage of stool | 3 (1.7) | 3 (1.7) | 2 (1.2) |
| Increased stool frequency | 7 (4.1) | 2 (1.2) | 1 (0.6) |
| Other reasons related to my UC | 2 (1.2) | 1 (0.6) | 0 |
| Pain in the genital area during sexual activity | 1 (0.6) | 1 (0.6) | 2 (1.2) |
| Unable to get or keep an erection | 1 (0.6) | 1 (0.6) | 0 |
| Worsening of abdominal pain during intercourse | 1 (0.6) | 1 (0.6) | 0 |

N, number of patients in the analysis population; n, number of patients in the specified category; SAA, sexual activity avoidance; UC, ulcerative colitis.

**Figure S1.** Improvement in bowel urgency severity (UNRS) from baseline to weeks 12 and 28

**Improvement**

BOCF, baseline observation carried forward; BU, bowel urgency; CFB, change from baseline; CI, confidence interval; SD, standard deviation; UNRS, Urgency Numeric Rating Scale.

**Reproduced from:** Danese S, Dignass A, Laharie D, et al. Mirikizumab is associated with rapid and sustained improvements in bowel urgency novel measures in moderately-to-severely active ulcerative colitis: 28-week results from the LUCENT-URGE trial. J Crohn's Colitis. 2026;20:jjag049. © The Author(s) 2026. Published by Oxford University Press on behalf of ECCO. This article is distributed under the terms of the Creative Commons Attribution License (CC-BY 4.0).
